# Targeting Hedgehog (Hh) Pathway for the Acute Myeloid Leukemia Treatment

**DOI:** 10.3390/cells8040312

**Published:** 2019-04-03

**Authors:** Toshiki Terao, Yosuke Minami

**Affiliations:** 1 Department of Hematology, National Cancer Center Hospital East, Kashiwa 277-8577, Japan; terao.toshiki@kameda.jp; 2 Division of Hematology/Oncology, Department of Internal Medicine, Kameda Medical Center, Kamogawa 296-8602, Japan

**Keywords:** Hedgehog pathway, acute myeloid leukemia, AML, leukemic stem cell, glioma, Smoothened, glasdegib, sonidegib

## Abstract

The Hedgehog (Hh) pathway, containing the Patched (PTCH) and Smoothened (SMO) multitransmembrane proteins, is the main regulator of vertebrate embryonic development. A non-canonical Hh pathway was recently observed in numerous types of solid cancers and hematological malignancies. Although acute myeloid leukemia (AML) is a common and lethal myeloid malignancy, the chemotherapy for AML has not changed in the last three decades. The Hh pathway and other intracellular signaling pathways are important for the tumor cells’ cycle or therapeutic resistance of AML cells. In this article, we will review the current trends in Hh pathway inhibitors for treating AML.

## 1. Introduction

Intracellular signaling pathways are considered as therapeutic targets for acute myeloid leukemia (AML). AML is a highly complex and heterogeneous disorder; conventional chemotherapy for patients with AML is partially effective, but not in all patients. The treatment for AML depends on the characteristics of each patient, such as age, performance status, and cytogenetic/molecular abnormalities. Systemic combination chemotherapy includes two phases: the induction phase and the consolidation of remission phase [1]. Older or unfit patients with AML cannot be administered intensive induction therapy because of their frailty. Thus, these patients may benefit from low-dose cytosine arabinoside (LDAC) or hypomethylating agents, such as azacitidine (AZA), which is a key drug for treating myelodysplastic syndrome (MDS). A recent phase III trial showed that AZA was effective in patients with relapsed/refractory AML [2]. For patients with an unfavorable risk or induction failure, allogeneic stem-cell transplantation is preferred [3,4,5,6]. Over the last three decades of administering cytotoxic induction therapy, we have seen that it is sometimes difficult to achieve remission, particularly in older or unfit patients.

In the 1990s, Dick et al. showed that CD34+ CD38-AML-initiating cells can migrate to the bone marrow (BM) and reconstitute human AML in immunodeficient mice [7]. This was demonstrated by the presence of leukemic stem cells (LSCs), which can proliferate, differentiate, and self-renew [8,9]. The BM environment in AML, which is reconstituted by LSCs, can be modified to suppress normal hematopoiesis while supporting leukemic cells [10]. This leukemic BM environment at initial diagnosis is related to the clinical outcome, relapse, or sustainment of complete remission (CR) for several years. Improving the understanding of LSCs and the BM environment can increase the number of therapeutic targets.

Some healthy elderly patients have clonal hematopoiesis of indeterminate potential (CHIP), which involves a mutation in a leukemia-associated gene at a variant allele frequency of >0.02 and an annual risk of developing a myeloid neoplasm [11,12]. These somatic leukemia-associated driver genes, such as DNA methyltransferase 3A (DNMT3A), ten-eleven translocation 2 (TET2), and isocitrate dehydrogenase 2 (IDH2), give rise to pre-LSC, which precedes the formation of fully transformed LSC [13,14,15]. These epigenetic changes are the earlier cooperating mutations in AML, rather than cytogenetic changes, such as those in the FMS-like tyrosine kinase 3 (FLT3) or Rat sarcoma (RAS) pathway [16,17]. As described above, AML is a highly complex disorder involving cytogenetic and epigenetic changes, and a new approach is needed to improve the treatment outcomes of AML patients.

In the following sections, we will describe the Hh-pathway activation and dysregulation in AML. We will summarize the various drugs used as Hh-pathway inhibitors, which are under clinical trials. We hope this review will be helpful for researchers in this field.

## 2. Hh Pathway Activation and Dysregulation

The Hh pathway was first discovered by Wieschaus and Nusslein-Volhard [18]. In the normal embryonic development, the Hh pathway is a well-conserved pathway and plays an important role for maintenance, regeneration, and redifferentiation of adult tissues. The secreted Hh proteins induce a series of cellular responses, such as survival, proliferation, differentiation, and fatal determination [19]. Proper Hh pathway at appropriate level requires the control of Hh ligand’s production, processing, secretion and transportation. In the canonical pathway, there are three types of Hh ligands in mammals (Sonic Hedgehog (SHH), Indian Hedgehog (IHH), and Desert Hedgehog (DHH)), with the pathway consisting of two receptors, a negative regulator (Patched (PTCH)) and a positive regulator (Smoothened (SMO)) [20]. Binding of Hh to PTCH1 derepresses the SMO, which leads to accumulation of SMO in the cilia and phosphorylation at the cytoplasmic end. This process mediates downstream signal transduction [21,22]. Glioma (GLI) zinc finger transcription factors (GLI1, GLI2, and GLI3) are activated by SMO activation, and activated GLI is translocated into the nucleus and binds the target DNA of the promoter to express specific genes, such as those encoding c-MYC, BCL2, and SNAIL [20,23,24,25]. Suppressor of Fused (SuFu) is also the element of the canonical Hh pathway and down-regulates GLI1-mediated target genes.

**Figure 1:**
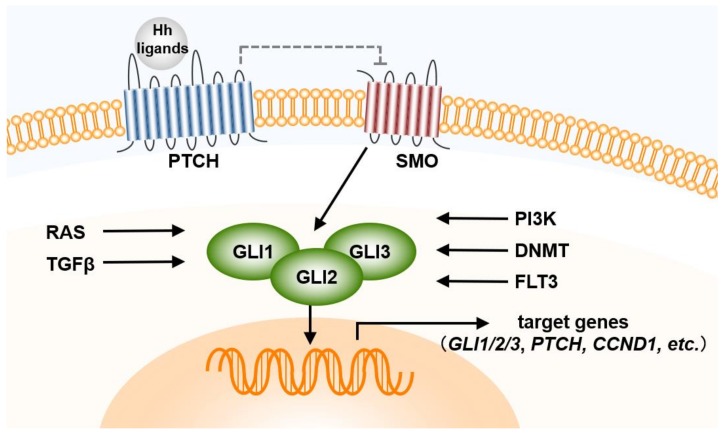
Hh pathway in AML cells. CCND, cyclin D; DNMT, deoxyribonucleic acid methyltransferase; FLT3, FMS-like tyrosine kinase 3; GLI, Glioma; Hh, Hedgehog; PI3K, phosphoinositide 3-kinase; PTCH, Patched; RAS, Rat sarcoma; SMO, Smoothened; TGF, transforming growth factor.

Although the expression of GLI1 reflects Hh pathway activity, several other proteins, such as RAS and transforming growth factor β, are the factors regulating GLI activity, regardless of the Hh pathway [26,27] (Figure 1). Abnormalities in this pathway lead to the dysregulation of cell regeneration, redifferentiation, or cell death, and depend on three mechanisms: Activation downstream of SMO resulting from genetic changes in PTCH, and autocrine or paracrine Hh pathways dependent on Hh ligands from BM stroma cells [28,29]. However, the mechanisms underlying the Hh pathway have not been completely clarified, particularly in cancer stem cells and LSCs. In addition to an important role in normal embryonic development and adult tissue homeostasis, aberrations in the Hh pathway affect the survival of hematological malignancy-related cells, such as chronic myeloid leukemia (CML) cells and multiple myeloma (MM) cells [30]. GLI-dependent Hh activation induced by the overexpression of GLI1 target genes, such as GLI1 and PTCH1, is observed in MM tumor cells. Therefore, understanding the regulatory mechanism of the Hh pathway leads to the development of new drugs and treatments for many Hh-dependent malignancies.

## 3. Hh Pathway in AML

The Hh pathway plays a role in the survival of AML patients. This pathway is related to the resistance of AML cells to drugs and radiotherapy, resulting in poor outcomes. GLI1 and the UDP-glucuronosyl transferase (UGT1A) family of enzymes are elevated in blasts with resistance to Ara-C [31]. UGT1As cause glucuronidation of many drugs, modifying their metabolism and attenuating their activities. GLI1 mRNA levels are elevated at relapse and decreased during the response. There is a clear correlation between GLI1 elevation and UGT1A activity, which results in a poor prognosis. Thus, drug resistance can be overcome by using a GLI1 inhibitor, but it remains a major problem for patients with AML.

Resistance to radiotherapy is another clinical problem. Total body irradiation with chemotherapy is the myeloablative regimen for allogeneic stem-cell transplantation, and radiotherapy is the part of an effective procedure in conditioning. The expression of Hh signaling proteins, including SMO and GLI1 in radiation-resistance myeloid leukemia cells, are higher than that observed in radiation-sensitive myeloid leukemia cells, suggesting that Hh pathway activation is related to radiotherapy resistance [32]. Another study revealed the expression of GLI-1 and SMO in AML cells, while no expression of Hh ligands was detected by quantitative polymerase chain reaction [33]. These results suggest that GLI and SMO can be activated in AML cells, regardless of the presence of Hh ligands. DHH plasma levels in AML patients and DHH mRNA levels in BM samples are higher than those in healthy individuals [33]. This is because a paracrine interaction may occur between leukemic cells and BM niche cells.

From the perspective of clinical outcomes, GLI2 expression has a negative influence on event-free survival (EFS), relapse-free survival, and overall survival (OS) and is correlated with the FLT3 mutation status. A single genetic mutation in FLT3-ITD causes gradual myeloid cell expansion as in a myeloproliferative neoplasm (MPN), but does not cause the full development of AML [34]. Although SMO is a canonical Hh pathway, constitutively active SMO cooperates with aberrant FLT3 activity to generate AML. Another protein, spleen tyrosine kinase (SKY), contributes to transform FLT3-ITD positive MPN into AML [35,36]. Recent data demonstrated that inhibition of GLI1 and SMO increase the activity of Ara-C for AML; thus, combination therapy for Hh pathway, SKY, and FLT3 is an acceptable and comprehensive therapeutic candidate and may improve clinical outcomes [31]. In AML, activation of the Hh pathway is a marker of poor prognosis; thus, it is desirable to suppress the Hh pathway.

As for MDS, the expression of DHH and SHH proteins was higher in the MDS cells than in the non-MDS cells (megaloblastic anemia cells) [37]. The expression of SMO was also increased in the BM of MDS patients, moreover, the patients with a higher expression of SMO showed significantly shorter 5-year EFS and 5-year AML evolution than those with a lower expression of SMO. In a murine model of MDS, there is a correlation between Hh/GLI1 activation and leukemic transformation, and SMO expression plays a role in the widespread expansion of immature myeloid cells, resulting in fatal AML [38,39]. A recent report showed that the Hh pathway does not play synergistic roles in hematopoietic stem cells because these functions could be masked by other signaling pathway, such as Notch and Wingless (Wnt), which can interact with the Hh pathway [40]. Studies are needed to determine the relationship between the Hh pathway and other intracellular signals.

## 4. Hh Pathway as a Therapeutic Target in AML

The SMO inhibitor, Vismodegib (GDC-0449), showed efficacy and was approved by the US Food and Drug Administration (FDA) for treating other non-hematological malignancies, such as basal cell carcinoma and basal cell nevus syndrome [41,42]. Because of the cross-talk between the Hh pathway and other intracellular signaling, monotherapy of the Hh pathway inhibitor did not show a sufficient effect on AML. However, combined administration of an Hh pathway inhibitor with other conventional drugs could involve two different mechanisms: direct action on the intracellular pathway and indirect action for overcoming the resistance of other drugs. Combination therapy could pave the way to further and innovative strategies for the pharmacological treatment of AML, not only to increase efficacy, but also to decrease toxicity of individual agents. Accordingly, several studies and clinical trials of the combination of an Hh pathway inhibitor and other agents, such as LDAC and AZA, have been conducted [43,44,45,46].

### 4.1. SMO Antagonist

#### 4.1.1. LDE225 (Sonidegib)

LDE225 is a specific SMO inhibitor and spreads to other multi-pathway through Hh-mediated regulation of target genes, which can inhibit insulin-like growth 1 receptor (IGF-1R), phosphoinositide 3-kinase (PI3K), Akt, and multidrug-resistance protein 1 (MRP1) pathway. In refractory primary AML cells and adriamycin-resistant AML cells, LDE225 increased cell apoptosis and the efficacy of adriamycin against tumor cells and lowered the expression of the targeted protein. For Adriamycin-treated AML cells, LDE225 suppressed tumor cell proliferation in an AML xenograft mouse model [47]. Using a combination of LDE225 and AZA, primary MDS and AML colony counts were reduced; the concentrations of LDE225 and AZA used were 2–4 and 1–2 μM, respectively, which is clinically feasible for AZA [46]. In a phase I/Ib trial, to determine the maximum tolerated dose (MTD) and best overall response with the use of LDE225 and AZA for myeloid malignancies including AML, the safety of LDE225 was observed and the response rate of SD was remarkably high, particularly in relapsed/refractory AML with a median follow-up of 14.5 months [48]. Common grade 3/4 hematological adverse effects (AEs) were thrombocytopenia (91%), followed by neutropenia (84%) and anemia (77%). The number of ClinicalTrials.gov Identifier was NCT02129101 and under examination.

#### 4.1.2. PF-04449913 (Glasdegib)

PF-04449913 (Glasdegib) is a selective SMO inhibitor and decreased the leukemia-initiation potential in AML cells in a serial transplantation mouse model [49]. The safety and tolerability of PF-04449913 in Japanese hematological malignancies has been demonstrated in a phase I study [50]. To evaluate the response of glasdegib, NANOG transcripts, which are associated with GLI-target genes, can act as biomarkers in glasdegib therapy [51]. In another phase-I safety and pharmacokinetics study of myeloid malignancies, 400 mg once daily was considered as the MTD, achieving a minor response (over 25% decrease from baseline in BM blasts) or better in more than 30% of AML patients [52]. Sixty percent of patients had grade 1–3 AEs, and 13% of patients had to decrease the dose because of treatment-related AEs. Subsequently, a combination 100 or 200 mg glasdegib once daily and LDAC or decitabine to unfit AML or high-risk MDS was generally well-tolerated in a phase-Ib study [45]. Based on these results, the recommended phase-II dose was determined as 100 mg once daily. A phase-II study to evaluate glasdegib from day 3 plus standard-induction chemotherapy (100 mg/m^2^ Ara-C on days 1–7 and 60 mg/m^2^ daunorubicin (DNR) on days 1–3 in a 28-day cycle) for untreated and fit AML or high-risk MDS patients revealed that 46.4% of patients achieved CR [43]. In all patients, the median OS was 14.9 months and most common grade 3/4 AEs were febrile neutropenia (FN) and anemia. Another phase II randomized study of 132 patients with unfit AML or high-risk MDS were treated with glasdegib (100 mg, oral, once daily) and LDAC (20 mg, subcutaneous, twice daily) or with LDAC monotherapy [44]. The glasdegib and LDAC arm resulted in an approximately 2-fold increase in OS as compared with results in the LDAC monotherapy arm (8.8 and 4.9 months, respectively). Because of the small number of patients with genetic mutations, no obvious conclusions regarding the association of specific genes with the response to therapy were confirmed. A randomized, double-blind, multicenter, placebo-control phase III trial to assess the efficacy of glasdegib in combination with induction-chemotherapy, or AZA, is currently underway (NCT03416179). Another phase II trial to determine the safety and tolerability of different drug combinations, such as glasdegib, gemtuzumab ozogamicin (GO, anti-CD33 monoclonal antibody), and PF-04518600 (OX40 agonist monoclonal antibody), is also ongoing (NCT03390296).

#### 4.1.3. GDC-0449 (Vismodegib)

GDC-0449 was first discovered from a library of small-molecule compounds and appeared to cause an objective response for locally advanced or metastatic basal-cell carcinoma [42]. GDC-0049 monotherapy exhibits efficacy toward AML cell lines, and a phase Ib trial for patients with relapsed/refractory AML administered 150 mg GDC-0449 once daily showed minimal clinical efficacy and safety [53]. A phase II trial to determine the response with ribavirin and GDC-0449 and/or decitabine for relapsed/refractory AML M4 or M5 in the FAB subtype (NCT02073838) was planned but not updated for two years.

#### 4.1.4. BMS-833923 (XL139)

BMS-833923 has a high-binding affinity to SMO receptors [54]. In a phase I trial, BMS-833923 with dasatinib improved the control of CML resistant to tyrosine kinase inhibitors [55]. A previous clinical trial, using BMS-833923 with lenalidomide or bortezomib (NCT00884546) to treat MM, was conducted; however, there is no clinical trial for AML or high-risk MDS to administer BMS-833923 that is underway.

### 4.2. GLI Antagonist

#### 4.2.1. GANT-61

GANT-61 blocks GLI1 function, decreases proliferation and clonogenic potential, and causes growth arrest and apoptosis in AML cells [39,56,57]. GLI-associated gene mutation and gene fusion, such as CBFA2T3-GLI-similar protein 2 (GLIS2), which is a fusion gene found in 17% of non-Down syndrome acute megakaryoblastic leukemia, were reported in pediatric AML, leading to a poor prognosis [58]. GANT-61 can induce apoptosis in AML cells with CBFA2T3-GLIS2 and decrease GLIS2-specific genes. In an FLT3-mutated mouse model, a combination GANT61 and sunitinib, a non-selective FLT3 inhibitor, prolonged the survival compared to each single agent [59]. Clinical trials of the GLI inhibitor are needed because GLI is regulated by not only the Hh pathway, but also by transcription factors.

We will show the representative clinical trials and their details of Hh-pathway-targeted agents in AML below (Table 1). As mentioned above, several Hh-pathway inhibitors are under clinical trials or developing; however, only one drug has now launched. FDA designated the application of glasdesib as a priority review and has approved it in combination with LDAC for newly diagnosed elderly (75 years old or older) or unfit AML patients on 21 November 2018. This decision was based on the NCT 01546038, which was a multicenter, open-label, randomized study.

## 5. Conclusions and Future Perspectives

In the last decade, numerous new agents for treating patients with AML, such as Hh pathway inhibitors, monoclonal antibodies, or other intracellular pathway inhibitors, have become available or are under clinical trials. No Hh pathway inhibitors other than glasdegib for AML or high-risk MDS have been approved, and their long-term toxicity profiles are unclear. In a dose escalation trial, the most frequent non-hematological AEs were associated with the gastrointestinal system, followed by alopecia and prolonged QTc. Among healthy donors treated with proton-pomp inhibitors, the same AEs were observed following the administration of 100 mg glasdegib daily [60]. Several SMO antagonists have been demonstrated to benefit patients with AML or high-risk MDS, whereas GLI antagonists are still in the preliminary stage of clinical trials. Recently, an isoflavone derivative, named as Glabrescione B (GlaB), was produced. GlaB is a small-molecule binding GLI1ZF and showed potential for reducing the growth of Hh/GLI-dependent tumor and cancer stem cells by interfering with its interaction with DNA [61,62]. In medulloblastoma cells, GlaB behaved as SMO and GLI antagonist, respectively, inhibiting the cell growth [61,63]. Similar mechanisms to inhibit Hh pathway are expected in AML cells.

A programmed death receptor-1 (PD-1) pathway inhibitor with CD8+ cytotoxic T-cell adoptive infusion can reduce the levels of human AML cells in an AML mouse model [64]. In gastric cancer cells, the Hh pathway modulates PD-L1 expression and, subsequently, promotes tumor proliferation [65]. Decreasing the expression of PD-L1, by using an Hh pathway inhibitor or a combination of an Hh pathway inhibitor and PD-1 blockade in AML cells or LSCs, may be effective. Another agent, such as venetoclax or CPX-351, which is a selective BCL-2 inhibitor, or in combination with Ara-C and DNR agents, respectively, may be useful for treating patients with unfit or secondary AML [66].

AML is a heterogeneous hematological malignancy. Numerous pathways including Hh pathway and genetic or epigenetic abnormalities may be correlated to the occurrence of AML, its pathological condition, and drug resistance. Future studies will provide an improvement of the outcomes of AML patients by using Hh-pathway inhibitors in combination with other novel agents.

## Figures and Tables

**Table 1:** Representative clinical trials of Hh-pathway-targeted agents in AML.

Agent	NCT Identified & Phase	Primary Outcomes Measures	Single or Combo Agents	Outcomes	Journal or Trial Status
LDE 225 (Sonidegib) (Novartis)	NCT 021229101 and Phase I/Ib	MTD and best overall response	LDE225 at 0–400 mg with AZA	MTD: 200 mg/day best overall response: 76% of R/R AML was SD and OS was 7.6 months.	Blood. 2017 130: 2629 (ASH abstract)
PF-04449913 (Glasdegib) (Pfizer)	NCT 02038777 and Phase I	First cycle DLT & safety	Single agent (25 mg, 50 mg, and 100 mg per day)	No DLT	Cancer Science. 2017, 108: 1628–1633
	NCT 00953758 and Phase I	First cycle DLT	Single agent (5 mg, 10 mg, 20 mg, 40 mg, 80 mg, 120 mg, 180 mg, 270 mg, 400 mg and 600 mg per day)	DLT determined. (each received 80 mg and 600 mg dose. *N* = 2) MTD: 400 mg per day	The Lancet Haematology. 2015, 2: e339–346
	NCT 01546038 and Phase Ib	MTD and RP2D	Glasdegib, in combo with LDAC (arm A), in combo with decitabine (arm B) and in combo with standard ICT (arm C)	No DLT in arm A and B, One DLT determined in arm C with grade 4 polyneuropathy. RP2D: 100 mg daily	Clinical Cancer Research. 2018, 24: 2294–2303
	NCT 01546038 and Phase II	CR with the final analysis defined as deaths in at least 40 of 60 patients ≥55 years old	Glasdegib, 100 mg daily with DNR and Ara-C	46.4% achieved CR of the 69 patients. In patients aged ≥55 years old, 40.0% achieved CR.	American Journal of Hematology. 2018, 93: 1301–1310
	NCT 01546038 and Phase II	OS	LDAC monotherapy (*N* = 41) or Glasdegib, 100 mg daily with LDAC (N = 84)	median OS: LDAC 4.9 months Glasdegib with LDAC 8.8 months	Leukemia. 2019, 33: 379–389
PF-04449913 (Glasdegib) (Pfizer)	NCT 03416179 and Phase III	OS	Invasive study: Arm A: Glasdegib + ′7 + 3′ Arm B: Placebo + ′7 + 3′ Non-intensive study: Arm C: Glasdegib + AZA Arm D: Placebo + AZA	No results available	Recruiting due for completion June 2021
	NCT 03390296 and Phase II	AE and CRc	Drug combinations: PF-04518600, Avelumab, AZA, Utomilumab, GO, Glasdegib	No results available	Recruiting due for completion December 2023
GDC-0449 (Vismodegib) (Genetech)	Phase Ib	Safety and Efficacy	Vismodegib, 150 mg daily	Safety: 97.4% of patients (including 84.2% with Grade ≥ 3) experienced at least one AEEfficacy: ORR was 6.1%.	British Journal of Haematology 11 September 2018 in online.
	NCT 02073838	ORR	Decitabine and Ribavirin with or without Vismodegib	No results available	This study is not verified in more than 2 years.

AE, adverse effect; AML, acute myeloid leukemia; ASH, American Society of Hematology; AZA, Azacitidine; CR, complete remission; CRc, Composite Complete response; DLT, dose-limiting toxicity; DNR, daunorubicin; GO, gemtuzumab ozogamicin; ICT, induction chemotherapy; LDAC, low-dose cytosine arabinoside; MTD, Maximal tolerated dose; NCT, National Clinical Trial; ORR, overall response rate; OS, overall survival; RP2D, recommended phase 2 dose; R/R, relapsed/refractory; SD, stable disease.

## References

[1] Wiernik P.H., Banks P.L., Case D.C., Arlin Z.A., Periman P.O., Todd M.B., Ritch P.S., Enck R.E., Weitberg A.B. (1992). Cytarabine plus idarubicin or daunorubicin as induction and consolidation therapy for previously untreated adult patients with acute myeloid leukemia. Blood.

[2] Itzykson R., Thepot S., Berthon C., Delaunay J., Bouscary D., Cluzeau T., Turlure P., Prebet T., Dartigeas C., Marolleau J.P. (2015). Azacitidine for the treatment of relapsed and refractory AML in older patients. Leuk. Res..

[3] Ganzel C., Manola J., Douer D., Rowe J.M., Fernandez H.F., Paietta E.M., Litzow M.R., Lee J.W., Luger S.M., Lazarus H.M. (2016). Extramedullary disease in adult acute myeloid leukemia is common but lacks independent significance: Analysis of patients in ECOG-ACRIN cancer research group trials, 1980–2008. J. Clin. Oncol..

[4] O’Donnell M.R., Tallman M.S., Abboud C.N., Altman J.K., Appelbaum F.R., Arber D.A., Bhatt V., Bixby D., Blum W., Coutre S.E. (2017). Acute myeloid leukemia, version 3.2017, NCCN clinical practice guidelines in oncology. J. Natl. Compr. Canc. Netw..

[5] Renneville A., Roumier C., Biggio V., Nibourel O., Boissel N., Fenaux P., Preudhomme C. (2008). Cooperating gene mutations in acute myeloid leukemia: A review of the literature. Leukemia.

[6] Slovak M.L., Kopecky K.J., Cassileth P.A., Harrington D.H., Theil K.S., Mohamed A., Paietta E., Willman C.L., Head D.R., Rowe J.M. (2000). Karyotypic analysis predicts outcome of preremission and postremission therapy in adult acute myeloid leukemia: A southwest oncology Group/eastern cooperative oncology group study. Blood.

[7] Lapidot T., Sirard C., Vormoor J., Murdoch B., Hoang T., Caceres-Cortes J., Minden M., Paterson B., Caligiuri M.A., Dick J.E. (1994). A cell initiating human acute myeloid leukaemia after transplantation into SCID mice. Nature.

[8] Bonnet D., Dick J.E. (1997). Human acute myeloid leukemia is organized as a hierarchy that originates from a primitive hematopoietic cell. Nat. Med..

[9] O’Brien C.A., Kreso A., Jamieson C.H. (2010). Cancer stem cells and self-renewal. Clin. Cancer Res..

[10] Kim J.A., Shim J.S., Lee G.Y., Yim H.W., Kim T.M., Kim M., Leem S.H., Lee J.W., Min C.K., Oh I.H. (2015). Microenvironmental remodeling as a parameter and prognostic factor of heterogeneous leukemogenesis in acute myelogenous leukemia. Cancer Res..

[11] Gibson C.J., Steensma D.P. (2018). New insights from studies of clonal hematopoiesis. Clin. Cancer Res..

[12] Steensma D.P. (2018). Clinical implications of clonal hematopoiesis. Mayo Clin. Proc..

[13] Chan S.M., Majeti R. (2013). Role of DNMT3A, TET2, and IDH1/2 mutations in pre-leukemic stem cells in acute myeloid leukemia. Int. J. Hematol..

[14] Jeong M., Park H.J., Celik H., Ostrander E.L., Reyes J.M., Guzman A., Rodriguez B., Lei Y., Lee Y., Ding L. (2018). Loss of Dnmt3a immortalizes hematopoietic stem cells in vivo. Cell Rep..

[15] Pandolfi A., Barreyro L., Steidl U. (2013). Concise review: Preleukemic stem cells: Molecular biology and clinical implications of the precursors to leukemia stem cells. Stem Cells Transl. Med..

[16] Jaiswal S., Fontanillas P., Flannick J., Manning A., Grauman P.V., Mar B.G., Lindsley R.C., Mermel C.H., Burtt N., Chavez A. (2014). Age-related clonal hematopoiesis associated with adverse outcomes. N. Engl. J. Med..

[17] Papaemmanuil E., Gerstung M., Bullinger L., Gaidzik V.I., Paschka P., Roberts N.D., Potter N.E., Heuser M., Thol F., Bolli N. (2016). Genomic classification and prognosis in acute myeloid leukemia. N. Engl. J. Med..

[18] Nusslein-Volhard C., Wieschaus E. (1980). Mutations affecting segment number and polarity in Drosophila. Nature.

[19] Ingham P.W., Nakano Y., Seger C. (2011). Mechanisms and functions of Hedgehog signalling across the metazoa. Nat. Rev. Genet..

[20] Varjosalo M., Taipale J. (2008). Hedgehog: Functions and mechanisms. Genes Dev..

[21] Stone D.M., Hynes M., Armanini M., Swanson T.A., Gu Q., Johnson R.L., Scott M.P., Pennica D., Goddard A., Phillips H. (1996). The tumour-suppressor gene patched encodes a candidate receptor for Sonic hedgehog. Nature.

[22] Taipale J., Beachy P.A. (2001). The Hedgehog and Wnt signalling pathways in cancer. Nature.

[23] Jia Y., Wang Y., Xie J. (2015). The Hedgehog pathway: Role in cell differentiation, polarity and proliferation. Arch. Toxicol..

[24] Sasaki H., Hui C., Nakafuku M., Kondoh H. (1997). A binding site for Gli proteins is essential for HNF-3beta floor plate enhancer activity in transgenics and can respond to Shh in vitro. Development.

[25] Scales S.J., de Sauvage F.J. (2009). Mechanisms of Hedgehog pathway activation in cancer and implications for therapy. Trends Pharm. Sci..

[26] Lauth M., Toftgard R. (2007). Non-canonical activation of GLI transcription factors: Implications for targeted anti-cancer therapy. Cell Cycle.

[27] Merchant M., Vajdos F.F., Ultsch M., Maun H.R., Wendt U., Cannon J., Desmarais W., Lazarus R.A., de Vos A.M., de Sauvage F.J. (2004). Suppressor of fused regulates Gli activity through a dual binding mechanism. Mol. Cell. Biol..

[28] Blotta S., Jakubikova J., Calimeri T., Roccaro A.M., Amodio N., Azab A.K., Foresta U., Mitsiades C.S., Rossi M., Todoerti K. (2012). Canonical and noncanonical Hedgehog pathway in the pathogenesis of multiple myeloma. Blood.

[29] Rubin L.L., de Sauvage F.J. (2006). Targeting the Hedgehog pathway in cancer. Nat. Rev. Drug Discov..

[30] Jagani Z., Dorsch M., Warmuth M. (2010). Hedgehog pathway activation in chronic myeloid leukemia. Cell Cycle.

[31] Zahreddine H.A., Culjkovic-Kraljacic B., Assouline S., Gendron P., Romeo A.A., Morris S.J., Cormack G., Jaquith J.B., Cerchietti L., Cocolakis E. (2014). The sonic hedgehog factor GLI1 imparts drug resistance through inducible glucuronidation. Nature.

[32] Li X., Chen F., Zhu Q., Ding B., Zhong Q., Huang K., Jiang X., Wang Z., Yin C., Zhu Y. (2016). Gli-1/PI3K/AKT/NF-kB pathway mediates resistance to radiation and is a target for reversion of responses in refractory acute myeloid leukemia cells. Oncotarget.

[33] Wellbrock J., Latuske E., Kohler J., Wagner K., Stamm H., Vettorazzi E., Vohwinkel G., Klokow M., Uibeleisen R., Ehm P. (2015). Expression of Hedgehog pathway mediator GLI represents a negative prognostic marker in human acute myeloid leukemia and its inhibition exerts antileukemic effects. Clin. Cancer Res..

[34] Lee B.H., Tothova Z., Levine R.L., Anderson K., Buza-Vidas N., Cullen D.E., McDowell E.P., Adelsperger J., Frohling S., Huntly B.J. (2007). FLT3 mutations confer enhanced proliferation and survival properties to multipotent progenitors in a murine model of chronic myelomonocytic leukemia. Cancer Cell.

[35] Lim Y., Gondek L., Li L., Wang Q., Ma H., Chang E., Huso D.L., Foerster S., Marchionni L., McGovern K. (2015). Integration of Hedgehog and mutant FLT3 signaling in myeloid leukemia. Sci. Transl. Med..

[36] Puissant A., Fenouille N., Alexe G., Pikman Y., Bassil C.F., Mehta S., Du J., Kazi J.U., Luciano F., Ronnstrand L. (2014). SYK is a critical regulator of FLT3 in acute myeloid leukemia. Cancer Cell.

[37] Xavier-Ferrucio J.M., Pericole F.V., Lopes M.R., Latuf-Filho P., Barcellos K.S., Dias A.I., de Melo Campos P., Traina F., Vassallo J., Saad S.T. (2015). Abnormal Hedgehog pathway in myelodysplastic syndrome and its impact on patients’ outcome. Haematologica.

[38] Gondek L.P., Lim Y., Makishima H., Wang Q., Maciejewski J.P., Aplan P.D., DeZern A.E., Matsui W. (2014). Aberrant Hedgehog pathway activity marks clinical MDS progression and accelerates leukemic transformation in vivo. Blood.

[39] Lau B.W., Huh K., Madero-Marroquin R., De Marchi F., Lim Y., Wang Q., Lobo F., Marchionni L., Smith D.B., DeZern A. (2019). Hedgehog/GLI1 activation leads to leukemic transformation of myelodysplastic syndrome in vivo and GLI1 inhibition results in antitumor activity. Oncogene.

[40] Gao J., Graves S., Koch U., Liu S., Jankovic V., Buonamici S., El Andaloussi A., Nimer S.D., Kee B.L., Taichman R. (2009). Hedgehog signaling is dispensable for adult hematopoietic stem cell function. Cell Stem Cell.

[41] LoRusso P.M., Rudin C.M., Reddy J.C., Tibes R., Weiss G.J., Borad M.J., Hann C.L., Brahmer J.R., Chang I., Darbonne W.C. (2011). Phase I trial of hedgehog pathway inhibitor vismodegib (GDC-0449) in patients with refractory, locally advanced or metastatic solid tumors. Clin. Cancer Res..

[42] Von Hoff D.D., LoRusso P.M., Rudin C.M., Reddy J.C., Yauch R.L., Tibes R., Weiss G.J., Borad M.J., Hann C.L., Brahmer J.R. (2009). Inhibition of the hedgehog pathway in advanced basal-cell carcinoma. N. Engl. J. Med..

[43] Cortes J.E., Douglas Smith B., Wang E.S., Merchant A., Oehler V.G., Arellano M., DeAngelo D.J., Pollyea D.A., Sekeres M.A., Robak T. (2018). Glasdegib in combination with cytarabine and daunorubicin in patients with AML or high-risk MDS: Phase 2 study results. Am. J. Hematol..

[44] Cortes J.E., Heidel F.H., Hellmann A., Fiedler W., Smith B.D., Robak T., Montesinos P., Pollyea D.A., DesJardins P., Ottmann O. (2018). Randomized comparison of low dose cytarabine with or without glasdegib in patients with newly diagnosed acute myeloid leukemia or high-risk myelodysplastic syndrome. Leukemia.

[45] Savona M.R., Pollyea D.A., Stock W., Oehler V.G., Schroeder M.A., Lancet J., McCloskey J., Kantarjian H.M., Ma W.W., Shaik M.N. (2018). Phase Ib study of glasdegib, a Hedgehog pathway inhibitor, in combination with standard chemotherapy in patients with AML or high-risk MDS. Clin. Cancer Res..

[46] Tibes R., Al-Kali A., Oliver G.R., Delman D.H., Hansen N., Bhagavatula K., Mohan J., Rakhshan F., Wood T., Foran J.M. (2015). The Hedgehog pathway as targetable vulnerability with 5-azacytidine in myelodysplastic syndrome and acute myeloid leukemia. J. Hematol. Oncol..

[47] Huang K., Ding B., Zhong Q., Jiang X., Li X., Wang Z., Meng F.Y. (2014). Hh/IGF-1R/PI3K/Akt/MRP1 Pathway Induce Refractory Acute Myeloid Leukemia and Its Targeting Therpy. Blood.

[48] Tibes R., Kosiorek H.E., Dueck A., Palmer J., Slack J.L., Knight E.A., Hashmi S.K., Bogenberger J.M., Zblewski D., Hogan W.J. (2017). Phase I/IB study of azacitidine and hedgehog pathway inhibition with Sonidegib (LDE225) in myeloid malignancies. Blood.

[49] Fukushima N., Minami Y., Kakiuchi S., Kuwatsuka Y., Hayakawa F., Jamieson C., Kiyoi H., Naoe T. (2016). Small-molecule hedgehog inhibitor attenuates the leukemia-initiation potential of acute myeloid leukemia cells. Cancer Sci..

[50] Minami Y., Minami H., Miyamoto T., Yoshimoto G., Kobayashi Y., Munakata W., Onishi Y., Kobayashi M., Ikuta M., Chan G. (2017). Phase I study of glasdegib (PF-04449913), an oral smoothened inhibitor, in Japanese patients with select hematologic malignancies. Cancer Sci..

[51] Kakiuchi S., Minami Y., Miyata Y., Mizutani Y., Goto H., Kawamoto S., Yakushijin K., Kurata K., Matsuoka H., Minami H. (2017). NANOG expression as a responsive biomarker during treatment with hedgehog signal inhibitor in acute myeloid leukemia. Int. J. Mol. Sci..

[52] Martinelli G., Oehler V.G., Papayannidis C., Courtney R., Shaik M.N., Zhang X., O’Connell A., McLachlan K.R., Zheng X., Radich J. (2015). Treatment with PF-04449913, an oral smoothened antagonist, in patients with myeloid malignancies: A phase 1 safety and pharmacokinetics study. Lancet. Haematol..

[53] Bixby D., Noppeney R., Lin T.L., Cortes J., Krauter J., Yee K., Medeiros B.C., Kramer A., Assouline S., Fiedler W. (2018). Safety and efficacy of vismodegib in relapsed/refractory acute myeloid leukaemia: Results of a phase Ib trial. Br. J. Haematol..

[54] Akare U.R., Bandaru S., Shaheen U., Singh P.K., Tiwari G., Singare P., Nayarisseri A., Banerjee T. (2014). Molecular docking approaches in identification of high affinity inhibitors of human SMO receptor. Bioinformation.

[55] Shah N.P., Cortes J.E., Martinelli G., Smith B.D., Clarke E., Copland M., Strauss L., Talpaz M. (2014). Dasatinib plus smoothened (SMO) inhibitor BMS-833923 in chronic myeloid leukemia (CML) with resistance or suboptimal response to a prior tyrosine kinase inhibitor (TKI): Phase I study CA180323. Blood.

[56] Long B., Wang L.X., Zheng F.M., Lai S.P., Xu D.R., Hu Y., Lin D.J., Zhang X.Z., Dong L., Long Z.J. (2016). Targeting GLI1 suppresses cell growth and enhances chemosensitivity in CD34 + enriched acute myeloid leukemia progenitor cells. Cell. Physiol. Biochem..

[57] Pan D., Li Y., Li Z., Wang Y., Wang P., Liang Y. (2012). Gli inhibitor GANT61 causes apoptosis in myeloid leukemia cells and acts in synergy with rapamycin. Leuk. Res..

[58] Masetti R., Bertuccio S.N., Astolfi A., Chiarini F., Lonetti A., Indio V., De Luca M., Bandini J., Serravalle S., Franzoni M. (2017). Hh/Gli antagonist in acute myeloid leukemia with CBFA2T3-GLIS2 fusion gene. J. Hematol. Oncol..

[59] Latuske E.M., Stamm H., Klokow M., Vohwinkel G., Muschhammer J., Bokemeyer C., Jucker M., Kebenko M., Fiedler W., Wellbrock J. (2017). Combined inhibition of GLI and FLT3 signaling leads to effective anti-leukemic effects in human acute myeloid leukemia. Oncotarget.

[60] Shaik N., Hee B., Wei H., LaBadie R.R. (2018). Evaluation of the effects of formulation, food, or a proton-pump inhibitor on the pharmacokinetics of glasdegib (PF-04449913) in healthy volunteers: A randomized phase I study. Cancer Chemother. Pharm..

[61] Infante P., Mori M., Alfonsi R., Ghirga F., Aiello F., Toscano S., Ingallina C., Siler M., Cucchi D., Po A. (2015). Gli1/DNA interaction is a druggable target for Hedgehog-dependent tumors. Embo J..

[62] Ingallina C., Costa P.M., Ghirga F., Klippstein R., Wang J.T., Berardozzi S., Hodgins N., Infante P., Pollard S.M., Botta B. (2017). Polymeric glabrescione B nanocapsules for passive targeting of Hedgehog-dependent tumor therapy in vitro. Nanomedicine.

[63] Berardozzi S., Bernardi F., Infante P., Ingallina C., Toscano S., De Paolis E., Alfonsi R., Caimano M., Botta B., Mori M. (2018). Synergistic inhibition of the Hedgehog pathway by newly designed Smo and Gli antagonists bearing the isoflavone scaffold. Eur. J. Med. Chem..

[64] Deng R., Fan F.Y., Yi H., Liu F., He G.C., Sun H.P., Su Y. (2018). PD-1 blockade potentially enhances adoptive cytotoxic T cell potency in a human acute myeloid leukaemia animal model. Hematology.

[65] Chakrabarti J., Holokai L., Syu L., Steele N.G., Chang J., Wang J., Ahmed S., Dlugosz A., Zavros Y. (2018). Hedgehog signaling induces PD-L1 expression and tumor cell proliferation in gastric cancer. Oncotarget.

[66] Lancet J.E. (2018). Is the overall survival for older adults with AML finally improving?. Best Pr. Res. Clin. Haematol..

